# Meta-analysis of Face Perception in Schizophrenia Spectrum Disorders: Evidence for Differential Impairment in Emotion Face Perception

**DOI:** 10.1093/schbul/sbae130

**Published:** 2024-08-13

**Authors:** Paige Mewton, Amy Dawel, Elizabeth J Miller, Yiyun Shou, Bruce K Christensen

**Affiliations:** School of Medicine and Psychology, College of Health and Medicine, The Australian National University, Canberra, Australia; School of Medicine and Psychology, College of Health and Medicine, The Australian National University, Canberra, Australia; School of Medicine and Psychology, College of Health and Medicine, The Australian National University, Canberra, Australia; School of Medicine and Psychology, College of Health and Medicine, The Australian National University, Canberra, Australia; Saw Swee Hock School of Public Health, National University of Singapore and National University Health System, Singapore; Lloyd’s Register Foundation Institute for the Public Understanding of Risk, National University of Singapore, Singapore; School of Medicine and Psychology, College of Health and Medicine, The Australian National University, Canberra, Australia

**Keywords:** differential deficit, cognition, memory, intelligence, ceiling effects, psychosis

## Abstract

**Background and Hypothesis:**

Schizophrenia spectrum disorders (SSD) are associated with face perception impairments. It is unclear whether impairments are equal across aspects of face perception or larger—indicating a differential impairment—for perceiving emotions relative to other characteristics (eg, identity, age). While many studies have attempted to compare emotion and non-emotion face perception in SSD, they have varied in design and produced conflicting findings. Additionally, prior meta-analyses on this topic were not designed to disentangle differential emotion impairments from broader impairments in face perception or cognition. We hypothesize that SSD-related impairments are larger for emotion than non-emotion face perception, but study characteristics moderate this differential impairment.

**Study Design:**

We meta-analyzed 313 effect sizes from 104 articles to investigate if SSD-related impairments are significantly greater for emotion than non-emotion face perception. We tested whether key study characteristics moderated these impairments, including SSD severity, sample intelligence matching, task difficulty, and task memory dependency.

**Study Results:**

We found significantly greater impairments for emotion (Cohen’s *d* = 0.74) than non-emotion face perception (*d* = 0.55) in SSD relative to control samples, regardless of SSD severity, intelligence matching, or task difficulty. Importantly, this effect was obscured when non-emotion tasks used a memory-dependent design.

**Conclusions:**

This is the first meta-analysis to demonstrate a differential emotion impairment in SSD that cannot be explained by broader impairments in face perception or cognition. The findings also underscore the critical role of task matching in studies of face perception impairments; to prevent confounding influences from memory-dependent task designs.

Schizophrenia spectrum disorders (SSD) are associated with face perception impairments.^1–7^ However, it is unclear whether these impairments are specific to (or larger for) face emotion perception or reflect global impairments in all facial judgments, including face identity, age, and gender.^1^ Studies have produced conflicting results which have been attributed to variations in study design.^1,8^ Although meta-analyses have attempted to quantify face perception impairments and how they are impacted by study features,^2,3^ they have omitted critical design components required to distinguish differential impairments from general cognitive-perceptual difficulties in SSD.^9,10^ Therefore, a refined meta-analysis is needed to determine whether impairments are larger for emotion than non-emotion face perception and identify study features that moderate the difference in impairments. Social cognitive and social functioning difficulties contribute substantially to the burden of illness in SSD^11–13^ and are associated with impairments in emotion face perception tasks.^14–16^ Thus, it is critical to determine whether emotion impairments are a specific characteristic of SSD or a secondary consequence of general cognitive-perceptual impairments, to better understand the mechanisms underlying SSD-related social cognitive difficulties and identify targets for treatment.

Individual studies measuring SSD-related face perception impairments have produced conflicting findings. The term “SSD-related impairments” throughout this article refers to lower task performance relative to community samples, which largely comprise of individuals without psychiatric diagnoses. While it is critical to identify areas where SSD samples perform statistically worse than community samples, we acknowledge that this approach does not capture instances where SSD samples demonstrate strengths or heightened functioning relative to community samples. The latter line of research is of equal importance, although outside of the scope of this paper. For example, impairments were found for both emotion and non-emotion tasks by Loughland et al^17^; for emotion but not non-emotion tasks by Bediou et al^18,19^; and for non-emotion but not emotion tasks by Bellack et al^20^. On balance, however, emotion impairments are observed more frequently (approximately 80% of studies^2^) than non-emotion impairments (approximately 30% of studies^21^). This pattern may reflect a larger emotion impairment^7^ or variation and limitations in study design.

A primary concern regarding prior meta-analyses and many individual studies is that they fail to directly compare the size of SSD-related impairments on emotion tasks with that of appropriate comparison tasks. This is crucial since people with SSD show general cognitive impairments^9^ and perform poorly relative to non-clinical samples across many cognitive tasks.^22–24^ This general cognitive impairment implies that SSD samples will likely perform worse than comparison samples on most tasks due to the cognitive processes involved. Therefore, to identify a differential emotion impairment, it is necessary to demonstrate significantly greater impairment on emotion tasks compared to non-emotion tasks with similar cognitive demands (see Chapman and Chapman^9,10,25^ for the differential deficit challenge). For example, Kohler’s^2^ meta-analysis found an SSD-related impairment in emotion perception but did not include a comparison task, so it cannot rule out the influence of global face perception or general cognition. In contrast, Chan et al’s^3^ meta-analysis assessed emotion and non-emotion face perception, and found SSD-related impairments for both task types (Cohen’s *d* for emotion tasks = −0.85; non-emotion tasks = −0.70). However, Chan et al did not test whether the impairment for emotion tasks was significantly larger than for non-emotion tasks, limiting the conclusions that can be drawn from their results. Additionally, prior meta-analyses have not adequately addressed key moderating factors, such as the demands or difficulty of experimental tasks.

## Key Sources of Study Design Variation

Inconsistent results in the literature are unsurprising given the substantial variability in study designs.^8^ Our second aim was to determine if differences in the size of emotion and non-emotion impairments are moderated by the key study characteristics reviewed below.

### SSD Severity

Studies vary in the SSD diagnostic samples they recruit, leading to differences in symptom severity and community dysfunction.^26^ Dimensional conceptualizations suggest SSD exists on a spectrum,^27^ with symptoms varying in magnitude rather than quality across diagnostic subcategories. Symptoms are most prominent in severe presentations (e.g., schizophrenia, schizoaffective disorder) and attenuated in milder presentations that are often without active psychosis symptoms (e.g., high trait schizotypy). Emotion perception impairments also appear to be dimensionally distributed in SSD, correlating with symptom severity,^1^ and being larger in samples with schizophrenia (e.g., Cohen’s *d* = 0.85^3^ to 0.91^2^) than milder SSD presentations (e.g., *d *= 0.48^28^). For non-emotion face perception, people with schizophrenia have moderate to large impairments which also correlate with symptom severity.^3^ However, evidence for non-emotion impairments in milder SSD presentations is lacking, with studies finding performance comparable to community samples.^29–31^ This lack of evidence for non-emotion impairments in milder SSD presentations may reflect under-powering of studies due to small effect sizes and small samples (e.g., Gee et al^32^ and Seiferth et al^33^).

Our meta-analysis included studies sampling the full range of SSD diagnoses to optimize power and investigate impairment profiles across the SSD spectrum. Consistent with a dimensional conceptualization,^27^ we hypothesized that the face perception impairment profile would be similar across SSD samples, but impairments would be smaller in milder SSD presentations (e.g., high schizotypy, early psychosis) compared to more severe presentations (i.e., schizophrenia, schizoaffective disorder). Consistent with smaller impairments, we hypothesized that the difference between emotion and non-emotion impairments would also be smaller in studies sampling low-severity compared to high-severity SSD.

### Intelligence Matching

SSD and comparison samples are often matched on measures of global intelligence (or years of education as a proxy) to control for SSD-related general cognitive impairments.^34,35^ However, this matching reduces meaningful variability between samples^36–38^ and may inadvertently recruit SSD or comparison samples with above- or below-average intelligence, respectively. This sampling bias, known as the matching fallacy, can dilute critical differences that would emerge with representative SSD and comparison samples, potentially misrepresenting true effects. Moreover, SSD symptom severity is negatively correlated with years of education^38^; as such, matching SSD and comparison samples on years of education would likely result in SSD samples with milder symptoms. Our meta-analysis tested whether intelligence matching moderates the difference between emotion and non-emotion face perception impairments, hypothesizing that the difference would be reduced when SSD and comparison samples were matched compared to unmatched for intelligence.

### Memory Dependency

Some face perception tasks call on non-face skills for successful completion. For example, face identification tasks often rely on memory. Typically, participants learn some target faces and are then asked to differentiate the targets from unlearned distractor faces (e.g., Warrington Recognition Memory Test^39^). The memory impairments associated with SSD^40^ may contribute to impaired performance on such tasks, irrespective of any impairment in face perception. Consistent with this, SSD-related impairments are more common in identity perception tasks that rely on memory.^21^ Our meta-analysis further interrogated if memory dependency moderates the difference between emotion and non-emotion face perception impairments, hypothesizing that the difference would be reduced when non-emotion tasks were memory-dependent compared to memory-independent.

### Task Difficulty

The difficulty of a task influences its sensitivity to reveal differences in ability between groups, potentially altering the apparent presence or size of group-related impairments.^9,10^ The impact of task difficulty is clearest in floor- and ceiling-effects. If a task is too easy or difficult, the score distributions of different samples will be compressed together, making it harder to distinguish true differences in the groups’ underlying abilities.^9,10^ Moreover, if emotion and non-emotion tasks are unequally difficult, differential impairments (e.g., for emotion perception) could be mistaken for global impairments (e.g., equal impairment for all face perception tasks).^10^ For example, comparing a close-to-ceiling emotion task with a more difficult non-emotion task may cause an emotion impairment to appear falsely small and equivalent to that for the non-emotion task. Indeed, commonly used emotion tasks are often criticized for producing close-to-ceiling accuracy in community samples.^41^ In contrast, identity perception is often measured by the Benton Test of Facial Recognition,^42^ which is routinely harder than the emotion face tasks used for comparison.^8,21^ Regrettably, the relative difficulty of tasks is rarely considered when measuring SSD-related face perception.^8^ In the present meta-analysis, we tested if task difficulty moderates the difference between emotion and non-emotion face perception impairments, hypothesizing that the difference would be reduced when tasks approached the ceiling.

## The Present Study

The present meta-analysis aimed to provide a direct and aggregated statistical comparison between emotion and non-emotion face perception impairments in SSD, previously missing from the literature. Our primary focus was on whether SSD-related impairments differed across task type (emotion vs non-emotion). We also investigated if several critical design variables might account for contradictory findings concerning a greater (differential) impairment for emotion tasks. In summary, these moderator variables were SSD severity, intelligence matching of samples, task memory dependency, and task difficulty.

We chose not to control for these variables via covariate analysis as this approach can obscure main effects by removing meaningful shared variance between main effects and covariates.^43^

The meta-analysis also provided a timely update in synthesizing this literature, with prior meta-analyses on the topic now over a decade old.^2,3^ We hypothesized that:

**H1:** SSD-related impairments would be significantly larger for emotion than non-emotion face perception tasks (i.e., a *differential impairment*).

**H2:** The differential emotion impairment would be moderated by:

a. SSD severity: the differential impairment would be smaller in low- compared to high-severity SSD samples.
b. intelligence matching: the differential impairment would be smaller when samples are matched compared to unmatched for intelligence.
c. memory dependency: the differential impairment would be smaller when non-emotion tasks are memory-dependent compared to memory-independent.
d. task difficulty: the differential impairment would be smaller when tasks were close-to-ceiling compared to not.

## Method

### Search Strategy

PubMed, PsychINFO, and Scopus databases were searched in January 2024 using terms relating to SSD, faces, and emotion (supplementary material S1). Eligibility criteria (supplementary material S2) required that articles:

1. included a sample with SSD and a control sample that were not a clinical group or relatives of people with SSD.
2. included a behavioral measure of accuracy for emotion *and* non-emotion face perception.
3. included stimuli that were static, whole images of human faces.
4. were published as a peer-reviewed full-text article in English.

### Screening and Data Extraction

**Fig. 1.**
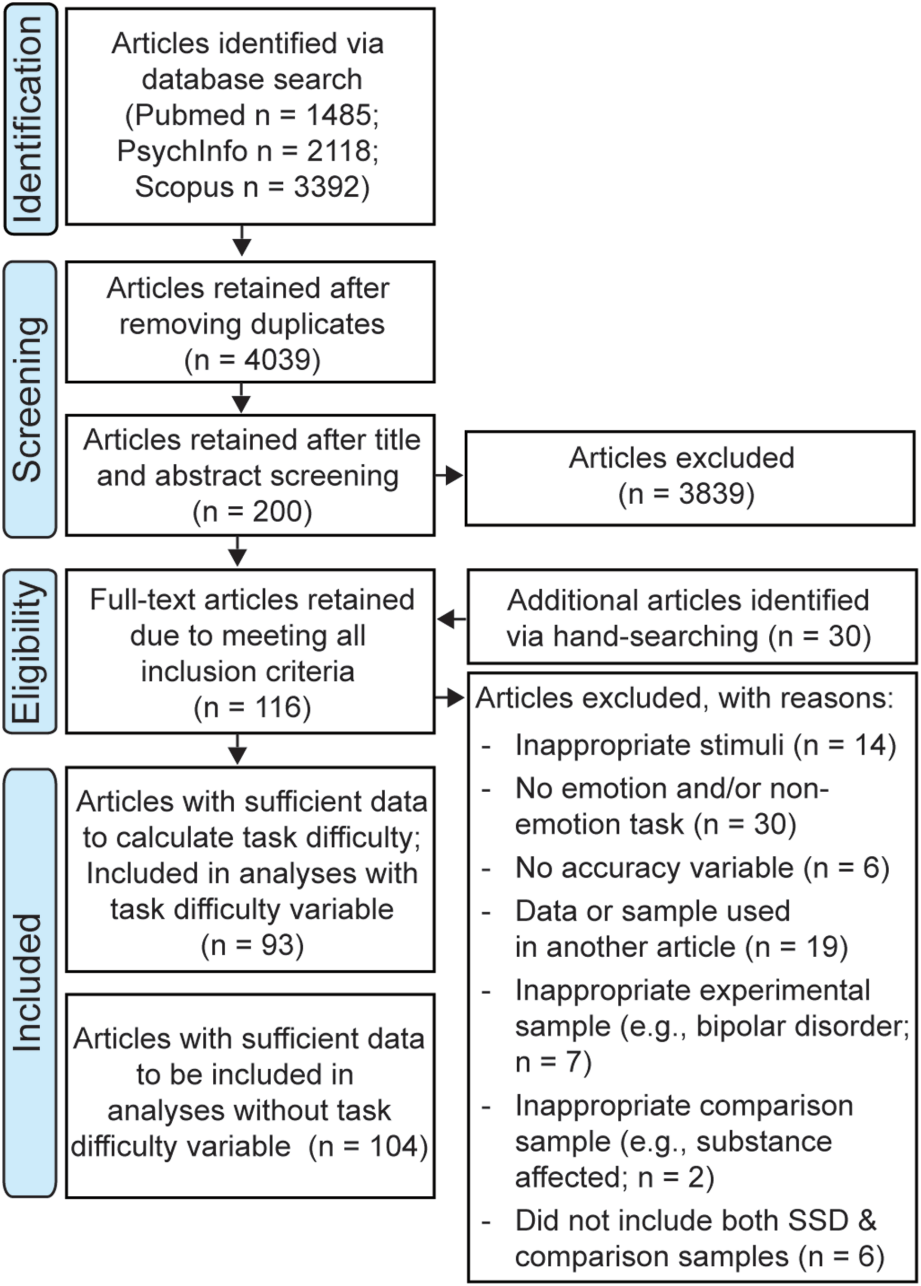
PRISMA flow chart of article search and screening process.

Figure 1 illustrates the eligibility screening process. Data were extracted by PM using Qualtrics (supplementary material S3). Web Plot Digitizer^44^ was used to extract numerical data from graphs. Authors were contacted at least twice to request critical data omitted from papers. To assess inter-coder reliability, 10% of articles were randomly selected and independently coded by EJM. Agreement between coders was high (*M* = 91.1%, *SD* = 6.3%, range = 78.7%–98.2%). Differences between coders were discussed and resolved.

Standardized mean differences between SSD and community sample task performance were calculated to quantify the size of SSD-related impairments. Cohen’s *d*^45^ was used as it is common in psychology and consistent with Chan et al’s^3^ approach. Where possible, means and SDs were extracted; otherwise, Cohen’s *d* was calculated from *F*- or *t*-values^46^ or extracted directly from articles. Larger Cohen’s *d* effect sizes indicated larger SSD-related impairments. Evidence of a differential impairment in emotion perception was indicated when there was a significantly larger effect size for emotion than non-emotion tasks.

If multiple papers reported overlapping samples (eg, Calkins et al^47^ and Pinkham et al^34^), the study with the largest sample size was included for analysis. Articles often included multiple emotion and/or non-emotion tasks, or provided data separately for different emotion categories within each task. Where possible (98% of data), effect sizes were extracted separately for separate tasks. When data was provided separately per emotion and a total task score was not available, a total score was estimated by combining the mean scores of individual emotions. Primary analyses test all emotions combined. Additionally, supplementary materials S4–S5 present the analysis of H1 across different emotion categories.

### Coding Moderator Variables

Table 1 lists the included articles^14,18–20,29,30,32,34,35,40,47–138^ and their moderator variables. Categorical moderators were coded at 2 levels. SSD severity was coded as high-severity (schizophrenia, schizoaffective disorders) and low-severity. The low-severity subset included all other diagnostic categories, which may increase heterogeneity but was necessary to achieve sufficient power for moderation analyses. Some articles reported combined high- and low-severity samples; these cases were conservatively coded as high-severity SSD to ensure low-severity group impairments were not amplified by a minority of high-severity participants (supplementary materials S6).

**Table 1.** Descriptive details of included articles and their moderator variables

Article	Task Type Effect Size Count					SSD sample	IQ matching	Memory-dependant non-emotion task used?	Close-to-ceiling task used?
	*Emotion*	*Identity*	*Gender*	*Age*	*Other*				
Addington & Addington, 1998	2	1	0	0	0	Schizophrenia	No	Both types	No
Andric et al., 2016	1	1	0	0	0	Other	No	No	No
Archer et al., 1992	1	2	0	0	0	Schizophrenia	Yes	Both types	Yes (Emotion, Identity)
Barkhof et al., 2015	1	1	0	0	0	Schizophrenia, Schizoaffective Disorder	Yes	Yes	Yes (Emotion, Identity)
Baudouin et al., 2002	3	3	0	0	0	Schizophrenia, Schizoaffective Disorder	No	Yes	Yes (Emotion, Identity)
Bediou et al., 2005	1	0	1	0	0	Schizophrenia	No	No	No
Bediou et al., 2007	1	0	1	0	0	Schizophrenia	No	No	No
Belge et al., 2017	1	0	1	1	0	Schizophrenia	Yes	No	Yes (Age, Emotion, Gender, Other)
Bellack et al., 1996	2	1	0	0	0	Schizophrenia, Schizoaffective Disorder	Yes	No	No
Bigelow et al., 2006	1	0	1	0	0	Schizophrenia, Schizoaffective Disorder, Brief Psychotic Disorder	No	No	Yes (Emotion, Gender)
Borod et al., 1993	2	1	0	0	0	Schizophrenia	No	No	Yes (Emotion, Identity)
Caldiroli et al., 2018	1	1	0	0	1	Schizophrenia	No	No	No
Calkins et al., 2010	1	1	0	0	0	Schizophrenia, Schizoaffective Disorder	No	Yes	No
Campanella et al., 2006	1	1	0	0	0	Schizophrenia, Schizoaffective Disorder	No	Yes	Yes (Emotion, Identity)
Chen et al., 2012	4	2	0	0	0	Schizophrenia, Schizoaffective Disorder	Yes	Yes	No
de Achaval, 2010	1	1	0	0	0	Schizophrenia	No	No	Yes (Emotion, Identity)
Derntl et al., 2009	1	0	0	1	0	Schizophrenia	Yes	No	No
Dickey et al., 2011	1	0	1	0	0	Schizotypal Personality Disorder	Yes	No	Yes (Emotion, Gender)
Doop & Park, 2009	1	0	0	0	0	Schizophrenia, Schizoaffective Disorder	Yes	No	Yes (Other)
Drusch et al., 2013	1	0	0	1	0	Schizophrenia	No	No	No
Drusch et al., 2014	1	1	0	0	0	Schizophrenia	No	No	No
Edwards et al., 2001	4	2	0	0	0	Schizophrenia, Schizoaffective Disorder, Schizophreniform Disorder, Other	Partial	No	Yes (Identity)
Erol et al., 2013	2	1	0	0	0	Schizophrenia, Schizoaffective Disorder, Schizophreniform Disorder, Other	Yes	No	No
Evangeli & Broks, 2000	1	4	0	0	0	Schizophrenia	Yes	Both types	Yes (Identity, Other)
Exner et al., 2004	1	1	0	0	0	Schizophrenia	Yes	No	No
Fakra et al., 2015	1	1	0	0	0	Schizophrenia	Yes	No	No
Feinberg et al., 1986	2	1	0	0	0	Schizophrenia	Yes	No	No
Frommann et al., 2013	1	1	0	0	1	Schizophrenia	Yes	No	No
Gee et al., 2012	2	0	2	0	0	Substance Induced Psychosis	No	No	Yes (Emotion, Gender)
Germine & Hooker, 2011	2	1	1	0	0	High Schizotypy	No	No	No
Gessler et al., 1989	3	0	0	3	0	Schizophrenia	No	No	No
Goghari et al., 2011	2	0	0	2	0	Schizophrenia	No	No	No
Goghari et al., 2017	1	0	0	1	0	Schizophrenia, Schizoaffective Disorder	Yes	No	No
Goghari, & Sponheim, 2013	1	0	0	1	0	Schizophrenia	Yes	No	No
Goldenberg et al., 2012	2	4	0	0	0	Schizophrenia, Ultra/clinical high risk of psychosis	Yes	Yes	No
Gur et al., 2002	1	0	0	1	0	Schizophrenia	No	No	No
Habel et al., 2000	3	0	0	3	0	Schizophrenia	Partial	No	Yes (Age, Emotion)
Habel et al., 2006	1	0	0	1	1	Schizophrenia, Schizoaffective Disorder, High Schizotypy, Other	Yes	No	No
Habel, Chechko et al., 2010	2	1	0	0	0	Schizophrenia	Yes	No	No
Habel, Koch et al., 2010	1	0	0	1	0	Schizophrenia	Yes	No	Yes (Emotion)
Hall et al., 2004	2	1	0	0	0	Schizophrenia	Yes	No	Yes (Emotion)
Hall et al., 2008	1	0	1	0	0	Schizophrenia	Yes	No	Yes (Emotion, Gender)
Heimberg et al., 1992	2	0	0	1	0	Schizophrenia	No	No	Yes (Emotion)
Hooker & Park 2002	1	2	0	0	0	Schizophrenia	Yes	Both types	Yes (Identity)
Horan et al., 2003	2	1	0	0	0	Schizophrenia	Yes	No	No
Johnston et al., 2010	1	1	0	0	0	Schizophrenia, Schizoaffective Disorder	Yes	No	Yes (Emotion, Identity)
Kerr & Neale, 1993	2	1	0	0	0	Schizophrenia	Yes	No	No
Kosmidis et al., 2007	2	1	0	0	0	Schizophrenia	Yes	No	No
Kucharska-Pietura et al., 2005	2	2	0	0	0	Schizophrenia	No	No	No
Kucharska-Pietura, Mortimer et al., 2012	1	1	0	0	0	Schizophrenia	No	No	Yes (Identity)
Kucharska-Pietura, Tylec et al., 2012	1	1	0	0	0	Schizophrenia	No	Yes	No
Kuo et al., 2018	1	1	0	0	0	Schizophrenia, Schizoaffective Disorder	No	Yes	No
Lee et al., 2020	1	1	0	0	0	Schizophrenia, Schizoaffective Disorder	No	Yes	No
Li et al., 2010	4	1	0	0	0	Schizophrenia	Yes	No	Yes (Identity)
Linden et al., 2010	1	3	0	0	0	Schizophrenia	No	Yes	No
Maat et al., 2020	1	1	0	0	0	Other	No	No	Yes (Emotion, Identity)
Mamah et al., 2021	2	1	0	0	0	Ultra/clinical high risk of psychosis	Yes	Yes	No
Martin et al., 2005	2	2	0	0	0	Schizophrenia	No	Yes	Yes (Emotion, Identity)
Martinez-Dominguez et al., 2015	1	2	0	0	0	Schizophrenia	Yes	Yes	No
Meijer et al., 2012	4	4	0	0	0	Schizophrenia, Schizoaffective Disorder, Schizophreniform Disorder, Brief Psychotic Disorder, Other	No	No	No
Mier et al., 2010	1	0	0	0	0	Schizophrenia	Yes	No	No
Mier et al., 2017	1	0	0	0	0	Schizophrenia	Yes	No	No
Modinos et al., 2020	1	1	0	0	0	Ultra/clinical high risk of psychosis	No	No	No
Mueser et al., 1996	2	1	0	0	0	Schizophrenia, Schizoaffective Disorder	No	No	No
Novic et al., 1984	1	1	0	0	0	Schizophrenia	Yes	No	No
Okada et al., 2015	2	2	0	0	0	Schizophrenia	No	No	No
Penn et al., 2000	4	2	0	0	0	Schizophrenia, Schizoaffective Disorder	No	No	Yes (Emotion)
Pinkham et al., 2008	1	2	0	0	0	Schizophrenia	Yes	Yes	No
Pinkham et al., 2019	1	1	0	0	0	Schizophrenia, Schizoaffective Disorder	No	No	No
Pomarol-Clotet et al., 2010	1	3	0	0	0	Schizophrenia	Yes	Both types	No
Poreh et al., 1994	1	1	0	0	0	High Schizotypy	Yes	Yes	Yes (Emotion, Identity)
Priyesh et al., 2021	2	0	1	0	0	Schizophrenia	Yes	No	Yes (Emotion)
Punchaichira et al., 2023	1	1	0	0	0	Schizophrenia	No	Yes	No
Quintana et al., 2011	1	1	0	0	0	Schizophrenia	Yes	No	Yes (Emotion, Identity)
Ramos-Loyo et al., 2009	1	1	0	0	0	Schizophrenia	No	Yes	Yes (Identity)
Ramos-Loyo et al., 2012	1	1	0	0	0	Schizophrenia	Yes	Yes	Yes (Identity)
Randers et al., 2020	1	1	0	0	0	Schizotypal Personality Disorder, Ultra/clinical high risk of psychosis	Yes	No	No
Rocca et al., 2009	5	1	0	0	0	Schizotypal Personality Disorder, Ultra/clinical high risk of psychosis	No	No	Yes (Emotion, Identity)
Sachse et al., 2014	1	1	0	0	0	Schizophrenia	Yes	No	No
Salem et al., 1996	2	1	0	0	0	Schizophrenia	Yes	No	No
Schneider et al., 1998	1	0	0	1	0	Schizophrenia	Yes	No	Yes (Age, Emotion)
Schneider et al., 2006	1	1	0	1	0	Schizophrenia	No	Both types	No
Schwartz et al., 2002	1	3	0	0	0	Schizophrenia, Schizoaffective Disorder	Partial	Yes	Yes (Identity)
She et al., 2017	2	2	0	0	0	Schizophrenia	Yes	No	Yes (Emotion)
Silver et al., 2003	1	1	0	0	0	Schizophrenia	No	Yes	No
Silver et al., 2005	2	1	0	0	0	Schizophrenia, Schizoaffective Disorder	Yes	Yes	No
Silver et al., 2009	3	1	0	0	0	Schizophrenia, Schizoaffective Disorder	No	Yes	No
Soria Bauser et al., 2012	3	3	0	0	0	Schizophrenia, Schizoaffective Disorder	No	Both types	Yes (Emotion, Identity)
Spilka & Goghari, 2017	1	0	0	1	0	Schizophrenia, Schizoaffective Disorder	Yes	No	No
Tripoli et al., 2022	1	1	0	0	0	First Episode Psychosis	No	No	No
Tylec et al., 2017	1	1	0	0	0	Schizophrenia, Schizoaffective Disorder	No	Yes	Yes (Identity)
van Rijn et al., 2011	1	1	0	0	0	Ultra/clinical high risk of psychosis	Yes	No	Yes (Identity)
van 't Wout et al., 2007	1	1	1	0	0	Schizophrenia, Schizoaffective Disorder, Schizophreniform Disorder	Yes	No	Yes (Emotion, Gender)
Villalta-Gil et al., 2013	2	1	1	0	0	Other	No	No	Yes (Emotion, Identity, Gender)
Walker, 1984	3	1	0	0	0	Schizophrenia	Yes	No	No
Watanuki et al., 2016	1	0	1	0	0	Schizophrenia	Yes	No	Yes (Emotion, Gender)
Whittaker et al., 2001	2	4	0	0	0	Schizophrenia	Yes	Both types	Yes (Emotion, Identity)
Wickline et al., 2012	1	1	0	0	0	Schizotypal Personality Disorder	Yes	No	No
Williams et al., 2007	2	1	0	0	0	High Schizotypy	No	No	No
Wynn et al., 2008	1	0	1	0	0	Schizophrenia	Yes	No	Yes (Emotion, Gender)
Wynn et al., 2013	1	0	1	0	0	Schizophrenia	No	No	Yes (Emotion, Gender)
Wynn et al., 2023	3	0	3	0	0	Schizophrenia, Schizoaffective Disorder, Schizophreniform Disorder, Delusion Disorder, Psychotic Disorder Not Otherwise Specified	Partial	No	Yes (Emotion, Gender)
Yagci et al., 2023	1	1	0	0	0	Schizophrenia	Yes	No	No
Yamada et al., 2009	1	1	0	0	0	Schizophrenia	Yes	No	No

*Note.* The Task Type Effect Size Count columns count the number of effect sizes for each task type, which may include different tasks, different samples completing each task, or a combination of the two. The IQ Matching column is labelled as “Partial” if some samples were matched for intelligence and others were not. If a close-to-ceiling task was used, the task type is listed in parentheses.

Intelligence matching was coded as matched or unmatched on a measure of global intelligence (or years of education as a proxy). Articles were coded as matched if the mean scores of SSD and community samples were not significantly different, regardless of whether matching was deliberate or incidental. Articles were coded as unmatched when samples’ scores differed significantly or if no intelligence/education measure was reported.

Tasks were coded as memory-dependent if they required participants to retain and recall previously learned information. Tasks were coded as memory-independent if they did not require memory for their completion. Memory-dependent tasks included primarily identity recognition tasks that required participants to memorize new faces, as well as tasks involving the recognition of famous faces from popular culture. Note, memory-dependent tasks always involved identity perception.

Task difficulty was coded as close-to-ceiling if the community sample scored ≥90% or not-at-ceiling if they scored below this criterion.

We defined ceiling tasks as those with ≥90% accuracy using the criteria that: (1) tasks must have high accuracy that is close to ceiling; and (2) accuracy threshold must retain enough studies to give no less than a 10:1 ratio between not-at-ceiling and close-to-ceiling effect sizes, as required for moderation analysis.^139^

We intended to code tasks as close-to-floor if the community sample scored ≤10% above chance performance; however, no tasks met this criterion. Task difficulty was also measured as a continuous variable via community sample accuracy (i.e., percentage of correct responses). Analyses including task difficulty were conducted on a subset of effect sizes as some articles provided insufficient data to calculate task difficulty (e.g., accuracy presented as raw scores without a total item number).

### Meta-analyses

Multilevel meta-regressions were conducted using mixed linear models in the Metafor package in R.^140^ Effect sizes were estimated using Restricted Maximum Likelihood models. Robust Variance Estimation^141^ was used to account for dependency among effect sizes caused by each article contributing multiple effects. Main and interaction effects were tested via *Q-*tests (comparable to χ² tests of association in general linear models) which identified whether significant variability among effect sizes could be explained by main or interaction effects.^142^ Study procedures followed PRISMA guidelines,^143^ although hypotheses and analysis plans were not preregistered. Data and analysis files are available at OSF (https://osf.io/jybqr/).^144^ Note, supplementary material S15 includes detailed descriptions of the tasks and samples, as well as all effect size and moderator data.

To test whether SSD-related impairments were significantly larger for emotion than non-emotion tasks (H1), we examined the main effect of task type. Most moderation effects were tested via interactions between task type and the relevant moderator variable (H2a: SSD severity; H2b: intelligence matching; H2d: task difficulty). The interaction between memory dependency and task type (H2c) could not be tested since emotion tasks were never memory dependent. Instead, articles were split into subsets that used memory-dependent vs memory-independent non-emotion tasks. When articles included both, memory-independent tasks were excluded from analyses so that no sample was included in both subsets. This approach aimed to balance the number of effect sizes provided by the 2 subsets. We then tested whether SSD-related impairments were larger for emotion than non-emotion tasks in the memory-dependent and memory-independent subsets separately.

Analyses were run twice: first as simple models including only the effects and interactions of interest and then as combined models including all possible predictor variables. Combined models tested whether the effect of interest accounted for a unique portion of variance not accounted for by other predictors. Combined models could not include both task type and memory dependency due to multicollinearity between them. There were no other multicollinearity issues among predictor variables. Combined models included fewer effect sizes due to missing data for task difficulty. Most often, simple and combined models produced the same results, so simple models are reported to optimize power. When simple and combined models produced conflicting results, both are reported.

Sensitivity analyses were conducted by rerunning analyses with influential outliers removed. Studentized residuals, Cook’s Distance, DFBETA values, and hat values^140^ were used to identify effect size and article level outliers that substantially influenced the models’ parameters (1%–7% of total cases in each model). In 1 instance, trend level results (*P* value = .05 to .10) became non-significant when outliers were removed so both findings are reported in text. Otherwise, results remained stable when outliers were removed, so we report models with outliers retained.

## Results

**Fig. 2.**
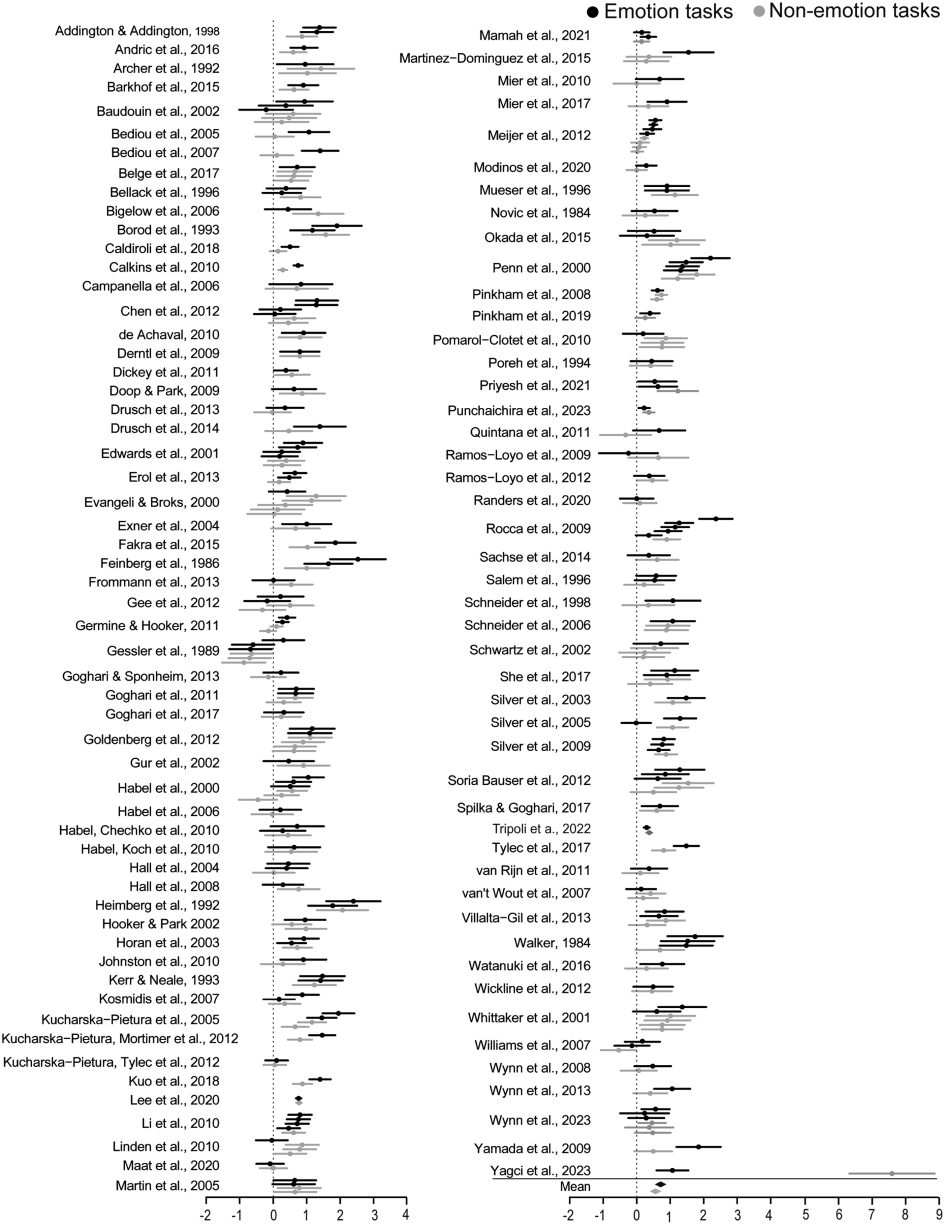
Forest plot. *Note.* Effect sizes are presented for each task and are grouped by article. Effects within each article may sample the same or different participants, or a mix of both. Error bars show 95% confidence intervals.

Table 2 shows the number of effect sizes, papers, and participants in each analysis and predictor variable category. Figure 2 shows effect sizes by article, for emotion and non-emotion impairments. Analyses revealed a moderate SSD-related impairment for all face tasks combined (*d* = 0.65, *p* < .001, 95% CI [0.56, 0. 74]). Effect sizes were significant for both emotion and non-emotion tasks at all levels of moderator variables, indicating there were SSD-related impairments regardless of task or study characteristics. This pattern is typical of SSD performance across cognitive tasks^22–24^ and demonstrates why significantly lower scores in SSD samples (relative to comparison samples) alone should not be interpreted as evidence for differential impairments. Therefore, we focus on the relative size of SSD-related impairments across task types, not the presence/absence of them. We found considerable heterogeneity among effect sizes (*I*^*2*^ range: 71%–85%, *p ≤* .001) indicating SSD-related impairments vary substantially across tasks. This heterogeneity warrants moderation analysis^145^ to determine which task features impact the size of impairments.

**Table 2.** Number of articles, effect sizes and participants in analyses and variable levels

Variable	*N* articles	*N* effect sizes	*N* SSD participants	*N* comparison participants
Primary Analyses Total	103	313	8036	6951
Task Type				
Emotion	103	163	7940	6883
Control	103	150	7973	6904
Identity	75	107	7128	6077
Age	14	20	450	408
Gender	15	18	623	605
Face Orientation	1	1	22	16
Eye Gaze Direction	2	2	42	42
Mixed	2	2	38	38
SSD Severity				
High-severity	90	272	5613	6245
Schizophrenia	69	204	3285	2982
Schizoaffective Disorder	3	7	69	616
Mixed Schizophrenia/Schizoaffective Disorder	18	48	2038	2740
Combined High- and Low-severity	5	12	157	150
Low-severity	15	41	1080	1978
High schizotypy	3	9	252	276
Schizotypal Personality Disorder	2	4	75	93
Early psychosis	4	10	134	734
High risk for psychosis	4	10	408	249
Substance-induced psychosis	1	4	20	14
Mixed	2	4	176	627
Intelligence Matching				
Matched	58	163	1730	1667
Not matched	49	150	6346	5353
Task Difficulty				
Task Difficulty Analyses Total	93	282	699	6863
Close-to-ceiling	42	87	1272	1144
Not-at-ceiling	81	195	6564	5870
Memory Dependency				
Memory-dependent subgroup	32	96	3535	2735
Memory-independent subgroup	72	207	4539	4211

*Note.* The number of articles and participants across moderator categories does not sum to the total number of articles or participants in the meta-analyses as articles and participants contributed to multiple categories. There are fewer effect sizes within the task difficulty analyses as some articles included insufficient detail to calculate task difficulty. The primary analyses total reflects the number of articles included in the primary analyses, not the number of articles extracted (n=104) as one article was included in task difficulty analyses and not the primary analyses due to overlapping participant groups across articles. The early psychosis SSD sample group includes samples defined as having schizophreniform disorder, attenuated psychosis syndrome and first-episode psychosis.

### H1: Are SSD-related Impairments Larger for Emotion vs Non-emotion Face Perception Tasks?

**Fig. 3.**
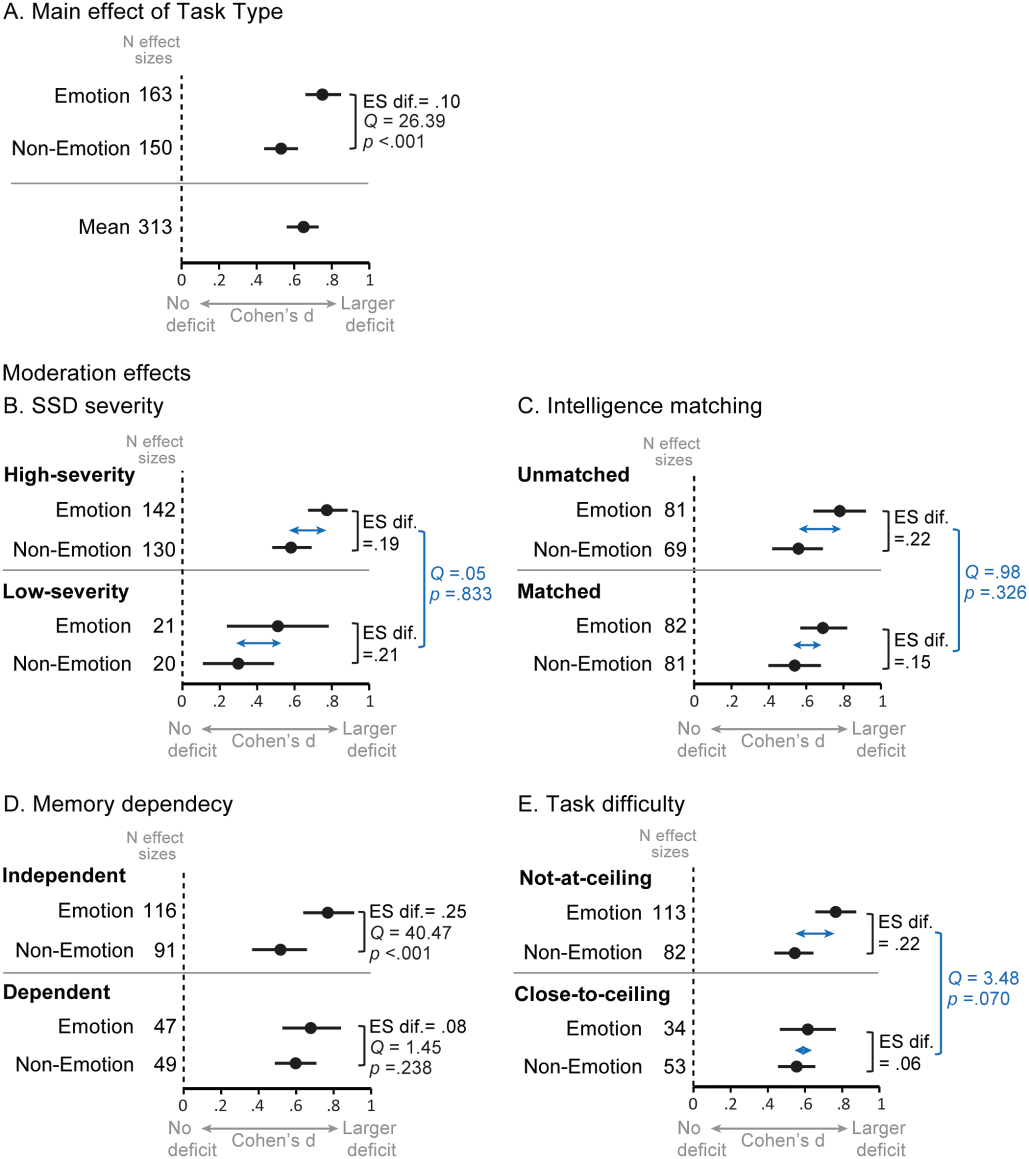
Effects of task type and moderator variables on SSD-related impairments. *Note.* All effect sizes are significantly different from zero. Error bars show 95% confidence intervals. The large confidence intervals in low-severity SSD samples reflect the small sample size and varied magnitude of deficits found across studies, likely due to sample diversity. In Fig 3A and 3D. the *Q* statistic tests the difference in effect size between emotion and non-emotion tasks. In Figs 3B., 3C., and 3E. the *Q* statistic tests the interaction between the moderator variable and task type.

Critically, we found SSD-related impairments were significantly larger for emotion (*d* = 0.74, *p* < .001, 95% CI [0.64, 0.83]) than non-emotion tasks (*d* = 0.55, *P* < .001, 95% CI [0.45, 0.64]), as demonstrated by a significant main effect of task type (*Q *= 26.39, *p* < .001, *k* = 313; figure 3A). This pattern indicates that people with SSD have a differential impairment in emotion face perception.

### H2a: Does SSD Severity Moderate the Size of the Differential Emotion Impairment?

When emotion and non-emotion tasks were combined, high-severity SSD samples showed significantly larger face perception impairments (*d* = 0.69, *p* < .001, 95% CI [0.59, 0.78]) than low-severity samples (*d* = 0.39, *p* = .001, 95% CI [0.20, 0.59]; *Q *= 8.16, *p* = .020, *k* = 313). Note, this difference approached significance in the combined model (*Q *= 5.25, *p* = .060, *k* = 282), indicating shared variance with other moderators. Critically however, there was no significant interaction between SSD severity and task type (*Q* = 0.046, *p* = .833, *k* = 313), indicating that the difference in magnitude between the emotion and non-emotion impairments was comparable for high-severity (*d* difference = 0.19) and low-severity SSD (*d* difference = 0.21; figure 3B). This finding demonstrates that the differential emotion impairment is a feature of SSD populations regardless of symptom severity.

### H2b: Does Intelligence Matching Moderate the Size of the Differential Emotion Impairment?

While face perception impairments were numerically smaller when SSD and comparison samples were matched for intelligence (*d* = 0.62, *p* < .001, 95% CI [0.51, 0.74]) than when they were not (*d* = 0.68, *p* < .001, 95% CI [0.54, 0.81]), this difference was not statistically significant (*Q* = 0.38, *p* = .539, *k* = 313). This pattern indicates that matching samples for intelligence may not substantially influence the magnitude of SSD-related face perception impairments.

Moreover, while the difference between emotion and non-emotion impairments was numerically smaller when samples were intelligence-matched (*d* difference = 0.15) compared to when they were not (*d* difference = 0.22; figure 3C), the interaction between intelligence matching and task type was not significant (*Q* = 0.98, *p* = .326, *k* = 313), indicating that the differential emotion impairment was not moderated by intelligence matching. Overall, these results suggest that matching samples for intelligence does not substantially influence the apparent size of the SSD-related differential emotion impairment.

### H2c: Does Memory Dependency in Non-emotion Tasks Moderate the Size of the Differential Emotion Impairment?

When considering only non-emotion tasks, impairments were numerically larger when measured with memory-dependent (*d* = 0.60, *p* < .001, 95% CI [0.49, 72]) compared to memory-independent tasks (*d* = 0.54, *p* < .001, 95% CI [0.42, 0.66]). However, this difference was not statistically significant (*Q* = 0.69, *p* = .413, *k* = 155), indicating that memory dependency may not substantially impact the magnitude of SSD-related impairments on non-emotion tasks.

Moreover, while impairments were numerically larger for emotion than non-emotion tasks in the memory-dependent subset (non-emotion tasks: *d* = 0.60, *p* < .001, 95% CI [0.49,0.71]; emotion tasks: *d* = 0.68, *p* < .001, 95% CI [0.53, 0.84]) as well as the memory-independent subset (non-emotion tasks: *d* = 0.52, *p* < .001, 95% CI [0.37, 0.66]; emotion tasks: *d *= 0.77, *p* < .001, 95% CI [0.64, 0.91]), this difference was statistically significant only for the memory-independent subset (*Q *= 40.47, *p* < .001, *k* = 207; figure 3D) and not for the memory-dependent subset (*Q *= 1.45, *p* = .238, *k* = 96). Thus evidence of a differential emotion impairment was absent for the memory-dependent subset. This finding was not explained by the type of non-emotion tasks used (i.e., the memory-dependent subset including only identity tasks while the memory-independent subset included gender, age, and other types of non-emotion tasks; supplementary materials S7). Overall, these results show that confounding task type with SSD-related memory impairments by comparing memory-independent emotion tasks with memory-dependent non-emotion tasks causes emotion and non-emotion impairments to appear equal.

### H2d: Does Task Difficulty Moderate the Size of the Differential Emotion Impairment?

Non-emotion tasks were close-to-ceiling more often than emotion tasks (23% of emotion and 39% of non-emotion tasks; χ² = 8.58, *p* = .003), indicating that non-emotion tasks are more often too easy and, therefore, at risk of underestimating SSD-related performance. This was further demonstrated via continuous task difficulty, as non-emotion tasks were significantly easier (*d* = 0.33, *p* < .018, 95% CI [0.06, 0.60]; see supplementary materials S8–S10 for analysis details).

Face perception impairments were smaller in close-to-ceiling tasks (*d* = 0.57, *p* < .001, 95% CI [0.47, 0.67]) when compared to not-at-ceiling tasks (*d* = 0.68, *p* < .001, 95% CI [0.59, 0.78]). This difference approached significance (*Q *= 3.51, *p* = .069, *k* = 282; *Q *= 3.00, *p* = .091, *k* = 282 when influential outliers were removed), providing tentative evidence that SSD-related face perception impairments may appear smaller when measured by close-to-ceiling tasks. Contrastingly, continuous task difficulty had no significant effect on the size of SSD-related impairments (*Q *= 0.23, *p* = .635, *k* = 282).

Similarly, the difference between emotion and non-emotion impairments appeared smaller when tasks were close-to-ceiling (*d* difference = 0.06) compared to not-at-ceiling (*d* difference = 0.22; figure 3E). The interaction between categorical task difficulty and task type was approaching significance (*Q *= 3.48, *p* = .070, *k* = 282), however, it reduced to a null effect when influential outliers were removed (*Q *= 2.11, *p* = .157, *k* = 276). There was no significant interaction between continuous task difficulty and task type (*Q *= 0.69, *p* = .411, *k* = 282; See supplementary materials S11 for Figure). Taken together, these results indicate that the differential emotion impairment is not moderated by task difficulty.

### Publication Bias Analysis

A residual funnel plot and extended multilevel meta-regression models^146^ were used to test for publication and time-lag biases (supplementary materials S12–S14). A significant time-lag bias was found (slope = −0.02, *p* < .01, 95% CI = [−0.03, −0.01]) indicating that effect sizes reduced over time. Correcting for time-lag bias produced slightly smaller effect sizes (i.e., unadjusted combined face perception Cohen’s *d* = 0.65; adjusted for time-lag bias = 0.64) and thus true SSD-related impairments may be marginally smaller than reported herein.

## Discussion

The present study provides the first direct, meta-analytic comparison of the size of emotion versus non-emotion face perception impairments in SSD. Our findings demonstrate that people with SSD have a robust, differential impairment in judging emotion from faces relative to other face perception abilities, refuting arguments that poor performance is caused by global impairments in face perception or other cognitive-perceptual processes.^21^ By establishing that impairments are significantly larger for emotion than non-emotion tasks, our results identify that people with SSD experience a specific and differential impairment in emotion perception. The differential emotion impairment was robust to several study design variables, including SSD severity, intelligence matching, and task difficulty. One critical exception, however, is the choice of comparison task. Using memory-dependent identity tasks disadvantages SSD samples due to their memory deficits^40^ and, in turn, inflates identity impairments and weakens the differential emotion impairment. This finding highlights the importance of matching demands across tasks to avoid confounding the results of 1 task with an area of cognitive vulnerability for SSD populations.

Overall, our findings indicate that the differential emotion impairment was robustly present regardless of SSD severity, intelligence matching, and task difficulty. High- and low-severity samples both demonstrated differential emotion impairments, consistent with the dimensional conceptualization of SSD.^27^ The presence of differential emotion impairments in samples with high trait schizotypy^29,107,128^ and symptomatically remitted schizophrenia^18,147^ suggests it is a trait-like feature of SSD persistent across states of wellness. Samples with low-severity SSD show considerable heterogeneity in impairment magnitude, consistent with this sample encompassing multiple populations along the SSD continuum. Researchers should continue investigating face perception in low-severity SSD samples so that future meta-analyses can investigate how impairments present across more discrete SSD presentations.

Neither the differential emotion impairment nor the size of face perception impairments in general were substantially impacted by intelligence matching. These null effects run contrary to warnings regarding the “matching fallacy”^148^ and call into question the true impact of intelligence matching. Relatives of people with SSD show milder cognitive impairments when matched with community samples for years of education.^149^ However, to the best of our knowledge, this effect has yet to be quantified in SSD samples. Future research should identify the empirical consequences of intelligence matching on face perception and cognitive impairments more broadly in SSD samples.

Our findings related to task difficulty simultaneously support the robustness of the differential emotion impairment and highlight the importance of avoiding close-to-ceiling tasks. Although close-to-ceiling tasks produced smaller SSD-related impairments, the differential emotion impairment remained even when measured by these less sensitive tasks. Notably, non-emotion tasks were more often close-to-ceiling (39%), indicating non-emotion impairments are likely underestimated. Despite this, the differential emotion impairment cannot be explained by underestimated non-emotion impairments as they remain when non-emotion tasks are not-at-ceiling *and* when both tasks are close-to-ceiling and, therefore, comparably lacking sensitivity. Emotion tasks are also often close-to-ceiling (23%), which may explain studies finding no differential emotion impairment if such studies included comparison tasks that were not at ceiling. Our finding—albeit preliminary—that close-to-ceiling tasks produce smaller SSD-related impairments emphasizes the importance of considering the difficulty of experimental tasks.

Surprisingly, continuous task difficulty had no observable relationship with SSD-related impairments. This lack of relationship could mean that task difficulty only impacts on the appearance of SSD-related impairments at the extremes (e.g., ceiling effects) or task difficulty alone insufficiently captures a task’s sensitivity to SSD-related impairments. A broader concept, task-discriminating power, describes how well a task can differentiate the scores of higher- and lower-performing samples.^9,10^ Discriminating power is impacted by other variables including reliability, item number, and item-level difficulty,^10^ in addition to the overall difficulty of the task. A more sophisticated measure of task-discriminating power may correlate with SSD-related impairments. This concept is compelling given that reliability varies considerably across face perception tasks.^150,151^ For example, if emotion tasks were systematically more reliable than non-emotion tasks, greater measurement precision could cause emotion impairments to appear larger. Researchers should consider task difficulty, reliability, and other aspects of discriminating power when selecting and matching their experimental tasks.

Given the SSD-related memory impairments,^40^ it is perhaps unsurprising that face perception abilities are confounded when measured by tasks with a substantial memory load. This raises the question: why would researchers use memory-dependent identity tasks at all? Perhaps for ecological validity, as real-life identity perception often requires memory in ways emotion perception does not.^152^ For example, our findings indicate people with SSD would have comparable difficulty recognizing an acquaintance in a crowd (ie, high memory load) and identifying someone’s emotion, whereas recognizing their boss would be easier (ie, lower memory load). When operationalizing the dual process of memory-dependent identity perception, researchers should emphasize that this confounds results to avoid overestimation of face-specific impairments. When identifying differential impairments in a discrete area of cognition, however, researchers should avoid tasks requiring multiple, distinct processes to prevent misattributing the cause of impairments.

### Clinical and Research Implications

Our findings have clinical implications for SSD populations. Robust evidence for a differential emotion impairment aligns with the assertion that “hot” or emotionally salient, reward-relevant aspects of cognition are more impaired in SSD than “cold” or logical, emotionally neutral aspects of cognition.^153,154^ People with SSD are also highly influenced by “hot,” emotionally salient stimuli^155–158^ which, combined with error in their perception of such stimuli, may contribute to their experience of positive symptoms (e.g., hallucinations). The differential emotion impairment also positions emotion perception difficulties as a specific characteristic of SSD that cannot be wholly accounted for by general cognitive-perceptual impairments. This finding justifies considering emotion perception impairments as a mechanism for difficulties in broader social cognitive processes (e.g., theory of mind, social problem solving) and thus a target for intervention. Researchers often speculate that these processes may be influenced by impaired emotion perception.^15,159^ While the current findings cannot speak to causality, they position emotion impairments as a strong candidate to pursue in treatment. For example, clinical interventions targeting emotion perception skills, such as emotion-specific cognitive remediation programs,^160^ now have stronger theoretical backing with the demonstration of a differential emotion impairment in SSD.

Our findings also offer guidance for future SSD-related face perception research. First, the shared impairment profile across high- and low-severity SSD samples supports recruiting more accessible, low-severity samples and generalizing findings across presentations. Although this may require larger sample sizes, our effect sizes can guide power analyses to avoid Type I errors. Second, as intelligence matching has no discernible impact on face perception impairments, findings from matched samples are a valid representation of true SSD-abilities—contrary to warnings about the “matching fallacy”^37,148^—and researchers can refrain from intelligence matching in the future. Third, researchers should carefully consider the demands of their tasks, which should be as similar as possible outside of the skill under investigation to avoid confounding results. This may require balancing ecological validity with confound minimization. For example, Face Recognition from the Amsterdam Neuropsychological Tasks^161^ (participants identify a previously shown, single target face from an array of 4) may be selected over the Warrington Recognition Memory Test^39^ (participants select which face from a pair they recognize from a previously shown list of 50 faces). Both tasks involve recognizing newly learned faces as is inherent to identity perception, but the Warrington test involves a high memory load rarely necessary in real-life settings (i.e., remembering 50 new faces after just 3 s of exposure). Finally, our results provide preliminary evidence that using close-to-ceiling tasks dilutes the magnitude of SSD-related impairments. Although the magnitude of the differential emotion impairment was not significantly impacted by task difficulty, figure 3E demonstrates that using close-to-ceiling tasks may still be problematic. Researchers would be prudent to use tasks with an optimal, moderate difficulty level^162^ until more is known about the impact of discriminating power on SSD-related face perception performance.

### Open Questions and Limitations

An important open question is whether the SSD-related impairment for non-emotion tasks reflects purely general cognitive impairments or a differential impairment in non-emotion face perception. Future work should investigate this question by comparing performance on non-emotion face tasks with non-face visual tasks. Guided by our findings regarding memory dependency, these tasks should be matched on memory demands and other cognitive-perceptual requirements to ensure performance scores capture the intended processes.

Another question is whether the scope of this meta-analysis has influenced results. It is possible that by including only articles with both emotion and non-emotion tasks, our meta-analysis includes mostly simple non-emotion tasks as comparison tasks. Articles that focus primarily on non-emotion face perception may use more complex non-emotion tasks and, consequently, may identify greater SSD-related impairments. Future research may wish to compare accuracy scores herein with a broader range of non-emotion face perception tasks from the literature to investigate this possibility.

A critical clinical direction is to explore which SSD symptoms are associated with emotion perception impairments. Existing evidence is varied. For example, Chan et al’s^3^ meta-analysis found that negative symptoms correlate with emotion impairments, whereas positive symptoms correlate with non-emotion impairments. In contrast, other studies have found positive symptoms correlate with emotion impairments but not non-emotion impairments.^163,164^ This inconsistency highlights the need to explore whether task (e.g., difficulty) or sample features (e.g., acute versus remitted diagnoses) moderate which SSD symptom profiles are most prone to emotion perception impairments.

We were limited by the constraints of subset analysis when investigating memory dependency as a moderator. There was a significant differential emotion impairment in the memory-independent subset and not the memory-dependent subset. However, without testing the interaction between memory dependency and task type we could not empirically test whether the magnitude of the differential emotion impairment differed significantly between the subsets. It is also possible that the memory-dependent subset showed no differential emotion impairment due to having fewer effects and therefore reduced power relative to the memory-independent subset. Despite these limitations, this was our best option for exploring the effect of memory dependency given variable constraints and available data.

Our coding of the intelligence matching variable was limited by the lack of information provided by articles. It was often unclear whether samples were deliberately or incidentally matched for intelligence. As such, both cases were coded as matched. However, samples that were incidentally matched were functionally indistinguishable from articles that did not measure intelligence (and thus were coded as not matched) but whose samples would have been incidentally matched if administered a measure. All cases were retained to maximize power, meaning there was an error in our variable of intelligence matching. Future research should endeavor to quantify the impact of intelligence matching on SSD-related impairments, for face perception and beyond.

## Conclusion

Our meta-analysis is the first to empirically demonstrate a differential emotion impairment in SSD, as evidenced by significantly larger impairments for emotion compared to non-emotion face perception. The differential emotion impairment is present regardless of key study characteristics including task difficulty, SSD severity, and whether experimental and control samples are matched on measures of intelligence. The differential emotion impairment is absent when non-emotion comparison tasks are memory-dependent, highlighting that the common practice of using memory-dependent identity perception tasks for comparison should be avoided. This practice confounds results by compounding face perception abilities with memory in the comparison task only, making for an unfair comparison with memory-independent emotion perception. Overall, our findings clarify that there are true, differential emotion face perception impairments in SSD that are not simply the result of general cognitive impairments. Rather, a subset of individual studies may not identify larger emotion impairments due to using poorly matched comparison tasks with additional cognitive demands. By identifying a differential emotion impairment, our findings position emotion impairments as a primary feature of SSD which can be targeted for treatment or investigated as a casual feature in functional difficulties.

## Supplementary Material

Supplementary material is available at https://academic.oup.com/schizophreniabulletin/.

sbae130_suppl_Supplementary_Material

sbae130_suppl_Supplementary_Data
