# Supplementary material for: Meta-analysis of Face Perception in Schizophrenia Spectrum Disorders: Evidence for Differential Impairment in Emotion Face Perception
Source: Schizophr Bull. 2024 Aug 13;51(1):17–36. doi: 10.1093/schbul/sbae130 (PMC11661959; doi:10.1093/schbul/sbae130)
Supplement: sbae130_suppl_Supplementary_Material [file sbae130_suppl_supplementary_material.docx]

### Supplementary Material

**S1. Search strategy**

Searches were conducted on 7th January, 2020 using the below search terms. We also hand-searched reference lists of all included articles and Google Scholar publication lists of authors who contributed to three or more included articles.

Table 1. Database search terms

| SSD terms | Face terms | Emotion terms |
| --- | --- | --- |
| Schizo*  Psychosis  Psychotic | Face  Faces  Facial  Expression* | Emoti*  Affect* |

***PubMed syntax (Results: 1485)***

( [Title/Abstract] ( ( schizo* OR psychosis OR psychotic) AND ( face OR facial or faces) AND ( emoti* OR affect OR expression)) NOT (meta analysis[Title] OR review[Title])

- Title/Abstract means that Pubmed is searching the title and abstract
- Fewer terms were included here due to the lack of proximity terms available on PubMed

***PsycInfo syntax (Results: 2118)***

( (psychotic or psychosis or schizo*) and (face or faces or facial or (recogni* adj5 affect*) or (recogni* adj5 emoti*) **or** (discrimina* adj5 affect*) or (discrimina* adj5 emoti*) or (identif* adj5 affect*) or (identif* adj5 emoti*) or (processing adj5 affect*) or (processing adj5 emoti*) or (percept* adj5 affect*) or (percept* adj emoti*)) and (( affect* adj5 face ) or ( affect* adj5 faces ) or (affect* adj5 facial) or emoti* or expression)).tw. not (review or (meta and analysis)).ti.

- tw. means that PsycInfo is searching the table of contents, title, abstract and key concepts.
- ti. Means that PsycInfo is searching the title.
- Adj5 is a proximity statement, which means that the preceding word must be found within 5 words of the subsequent word to be included in the results.

***Scopus (Results: 3392)***

( TITLE-ABS-KEY (schizo* OR psychosis OR psychotic) AND TITLE-ABS-KEY ( face OR facial OR (discrimina* W/5 affect*) OR (discrimina* W/5 emoti*) OR (identif* W/5 affect*) OR (identif* W/5 emoti*) OR (percept* W/5 affect*) OR (percept* W/5 emoti*) OR (processing W/5 affect*) OR (processing W/5 emoti*) OR (recogni* W/5 affect*) OR (recogni* W/5 emoti*)) AND TITLE-ABS-KEY ( emoti*  OR  expression  OR  (affect* W/5 face ) OR ( affect* W/5 facial )) AND NOT (TITLE (meta AND analysis) OR (review))

- TITLE-ABS-KEY means that Scopus is searching the title, abstract and keywords.
- TITLE means that Scopus is searching the title.
- W/5 is a proximity term, and means that the preceding words must be found within 5 words of face to be shown as a result.
- Meta-analysis and review terms were excluded here as 31 meta-analyses and reviews were included after using the search filter function to exclude review papers
- “Faces” not included as Scopus includes plurals automatically

**S2. Eligibility criteria**

The inclusion criteria were:

1. Studies included a sample with SSD (including Schizophrenia, Schizoaffective, or Schizophreniform Disorders; Schizotypal or Schizoid Personality Disorders; or brief intermittent or substance-induced psychosis; or subclinical symptoms including ultra-high or clinical risk of psychosis, or high trait Schizotypy [defined by the top 10% of scorers on measures of schizoptypy]) and a non-clinical control sample without SSD.
2. Studies included a control sample that were *not* recruited on the basis of being relatives of people with SSD, or having mental health diagnoses or cognitive impairments likely to influence face perception (i.e., brain injuries, pervasive developmental disorders [e.g., autism], current substance abuse, or subnormal [-2 SD] intelligence).
3. Studies could recruit participants of any age, as long as the control sample were similarly matched.
4. Studies included a behavioral measure of accuracy (e.g., number or percentage of correct responses; signal detection theory measures of sensitivity; difference between valence rating score and correct score) for emotion face perception (e.g., emotion labelling, discrimination or valence rating; judgement of emotion intensity; matching faces by expressed emotion) and non-emotion face perception (e.g., identity matching or recognition; age or gender labelling; matching eye-gaze or face direction). Indirect or partial measures of performance accuracy were excluded (e.g., hit rate without false alarm rate; ratio of the number of targets counted to the number of targets present in an array).
5. Studies used static images of whole, upright human faces (e.g., not stimuli that were dynamic, inverted, audio, body posture, or avatars).
6. Studies were presented as full-text, published in peer-reviewed journals, and available in English.
7. Studies that provided sufficient data to calculate an effect size statistic comparing the accuracy of SSD and control samples on emotion and non-emotion face perception tasks.
8. Studies where effect size data was not adjusted to remove variation accounted for by SSD-related variables (e.g., accuracy data adjusted for participant intelligence scores; F-statistics from ANOVAs using accuracy on other face-processing tasks as a covariate).

**S3. Data extraction form**

See S3 spreadsheet.

**S4. Is H1 influenced by emotion type?**

| **Table S5. Effect size data for emotion categories** | | | | | | |
| --- | --- | --- | --- | --- | --- | --- |
| **Task** | | ***N* effect sizes** | **Effect Size Estimate and SE** | **95% CI** | | **Comparison with non-emotion tasks (p-value)** |
| Non-emotion Tasks | | 150 | .77 (.11) | .57 - .98 |  | |
| Emotion Tasks | |  |  |  |  | |
| Positive valence | | 4 | 1.32 (.23) | .34 – 2.31 | | .183 |
| **Combined** | | **39** | **1.01 (.11)** | **.80 – 1.22** | | **<.001** |
| Negative valence | | 4 | .98 (.13) | .42 – 1.54 | | .243 |
| Shame | | 3 | .89 (.11) | .37 – 1.40 | | .185 |
| **Fear** | | **50** | **.87 (.12)** | **.64 – 1.10** | | **.039** |
| Sad | | 37 | .84 (.12) | .60 – 1.08 | | .261 |
| Anger | | 48 | .82 (.13) | .57 - 1.07 | | .528 |
| Surprise | | 17 | .80 (.14) | .51 – 1.09 | | .792 |
| Disgust | | 21 | .79 (.14) | .51 – 1.06 | | .899 |
| Interest | | 2 | .77 (.11) | .55 – 1.00 | | .994 |
| Neutral | | 28 | .74 (.13) | .49 - .99 | | .665 |
| Happy | | 59 | .72 (1.2) | .49 - .95 | | .340 |
| Contempt | | 3 | .64 (.21) | -.23 – 1.50 | | .585 |
| *Note.* Effect sizes for emotion tasks are sorted from largest to smallest impairment. Emotion subtasks are bolded if they produced significantly larger impairments when compared to non-emotion tasks. | | | | | | |

We investigated whether SSD-related differential emotion impairments persisted for all emotion subcategories; that is, whether emotion impairments are larger than non-emotion impairments (H1) across all types of emotion. To do so, we ran a model comparing impairments for emotion tasks split by the type of emotion used with that of all non-emotion comparison tasks. Findings are presented in Table S6. Data should be interpreted with caution, however, due to the small number of effect sizes included in most emotion subcategories relative to the substantial number of non-emotion effect sizes. Fear and combined emotion tasks (i.e., where data was provided for the whole task only, which included multiple emotions) produced significantly larger impairments than non-emotion tasks, consitant with a differential emotion impairment. Impairments for most other emotion subcategories (i.e., all but interest, neutral, happy, and contempt) were numerically larger than for non-emotion tasks, however, these differences were not significant likely due to large variation amongst fewer effect sizes.

**S6. Treatment of combined severity SSD**

Moderators were only included if the ratio of the smallest to the largest number of effect sizes across moderator levels exceeded 1:10^1^. Therefore, the 11 effect sizes using combined high- and low-severity SSD samples (vs. 247 from high-severity samples) could not comprise a separate moderator level. Instead, we ran two sets of analyses: (1) excluding the mixed-severity group and (2) including them in the high-severity SSD group. Two papers with combined severity samples included a minority of low-severity SSD participants, so they were added to the high-severity group. All other studies were roughly balanced and were added to the high-severity SSD group as a conversative approach. The primary results did not differ, so the latter is presented in our results.

**S7. Re-analysis of H2c with only identity perception comparison tasks**

As only identity tasks were memory-dependent (i.e., never age, gender or other non-emotion tasks), we tested whether the moderating effect of memory dependency could be explained by the type of non-emotion tasks used. For example, if identity tasks produced larger impairments than age and gender tasks, this would cause larger non-emotion impairments (and therefore, a reduced differential emotion impairment) in the memory-dependent subset. To test this, we replicated H2c analyses while including just identity tasks as non-emotion tasks. All primary results were the same supporting the conclusion that memory-dependency moderates the differential impairment in emotion perception.

**S8. Continuous task difficulty analysis**

Task difficulty was also measured as a continuous variable via the percentage accuracy score of the control sample. When looking across all included tasks, difficulty was slightly lower for emotion tasks (*M* = 82.6%, SD = 13.1%) than non-emotion tasks (*M* = 85.4%, SD = 12%). Distributions of task difficulty are shown in Figure S8.

To test whether task difficulty differed significantly between emotion and non-emotion tasks, we calculated the standardised mean difference (Cohen’s *d*) between control sample performance on emotion versus non-emotion tasks. One effect size was calculated per study. When a study measured more than one emotion or non-emotion task, scores were combined to produce a single score. A meta-analysis was run to test whether task difficulty was significantly higher for emotion versus non-emotion tasks. A positive effect size indicated higher task difficulty (i.e., lower control sample scores) for emotion tasks. The meta-analysis was conducted as per the main text, except that Robust Variance Estimation was not required as each paper only contributed to only one effect size (no need to account for dependency amongst effects). Note that fewer studies were included in this analysis than those including task difficulty as a moderator due to missing data required to calculate standardised mean difference between control samples across tasks (i.e., no control sample SD).

Studies typically used emotion tasks that were significantly more difficult than non-emotion tasks (d = 0.33, *p* = .018, 95% CI [.06 - .60], k = 86). As average task difficulty for both task types were high (i.e., nearing close-to-ceiling), non-emotion tasks were significantly more likely to be problematically easy. The forest plot in Fig. S9 shows effect sizes across articles.

**
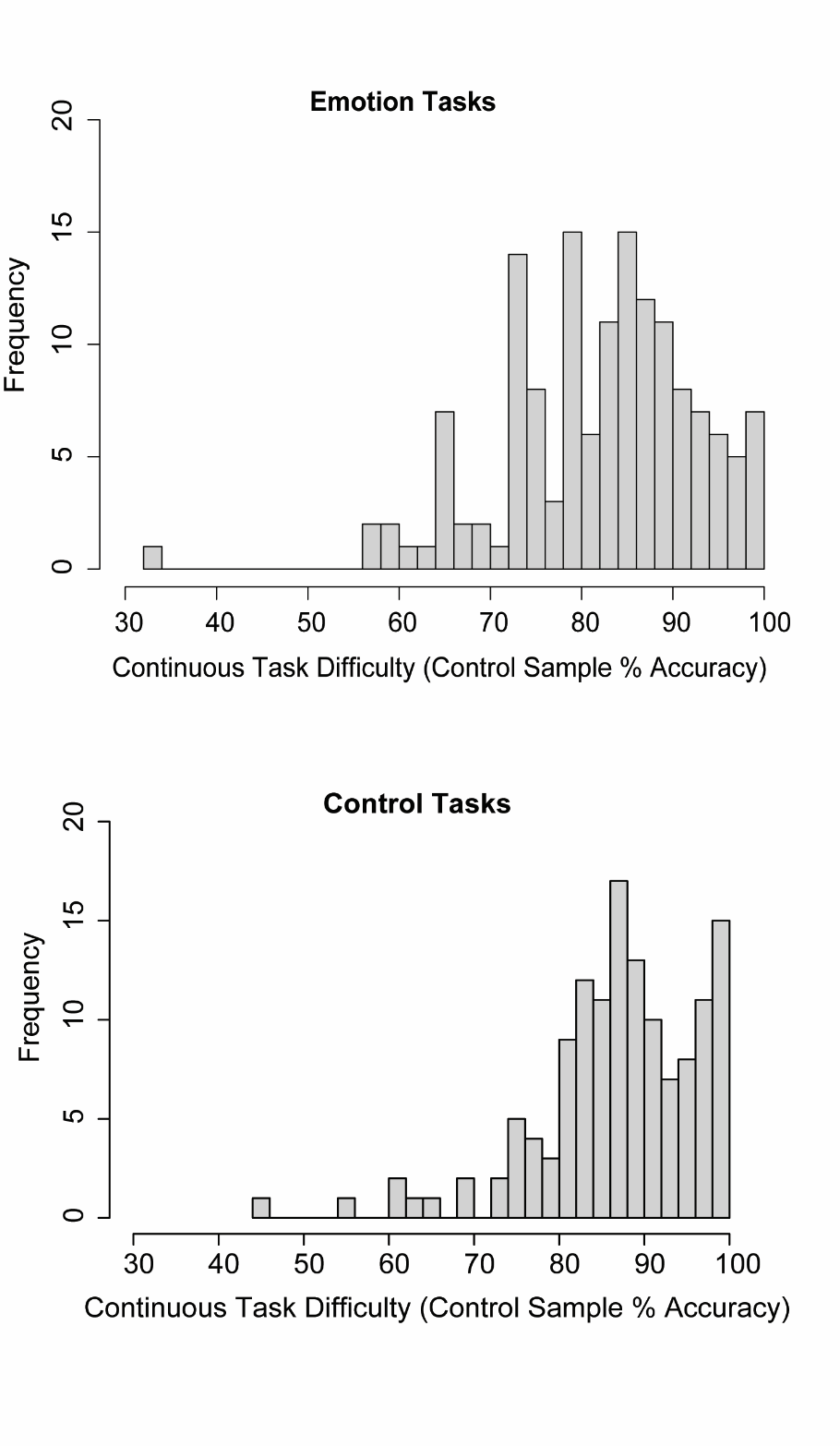
Figure S9. Distributions of continuous task difficulty**

**S10. Forest plot for emotion vs. non-emotion task difficulty**

**_
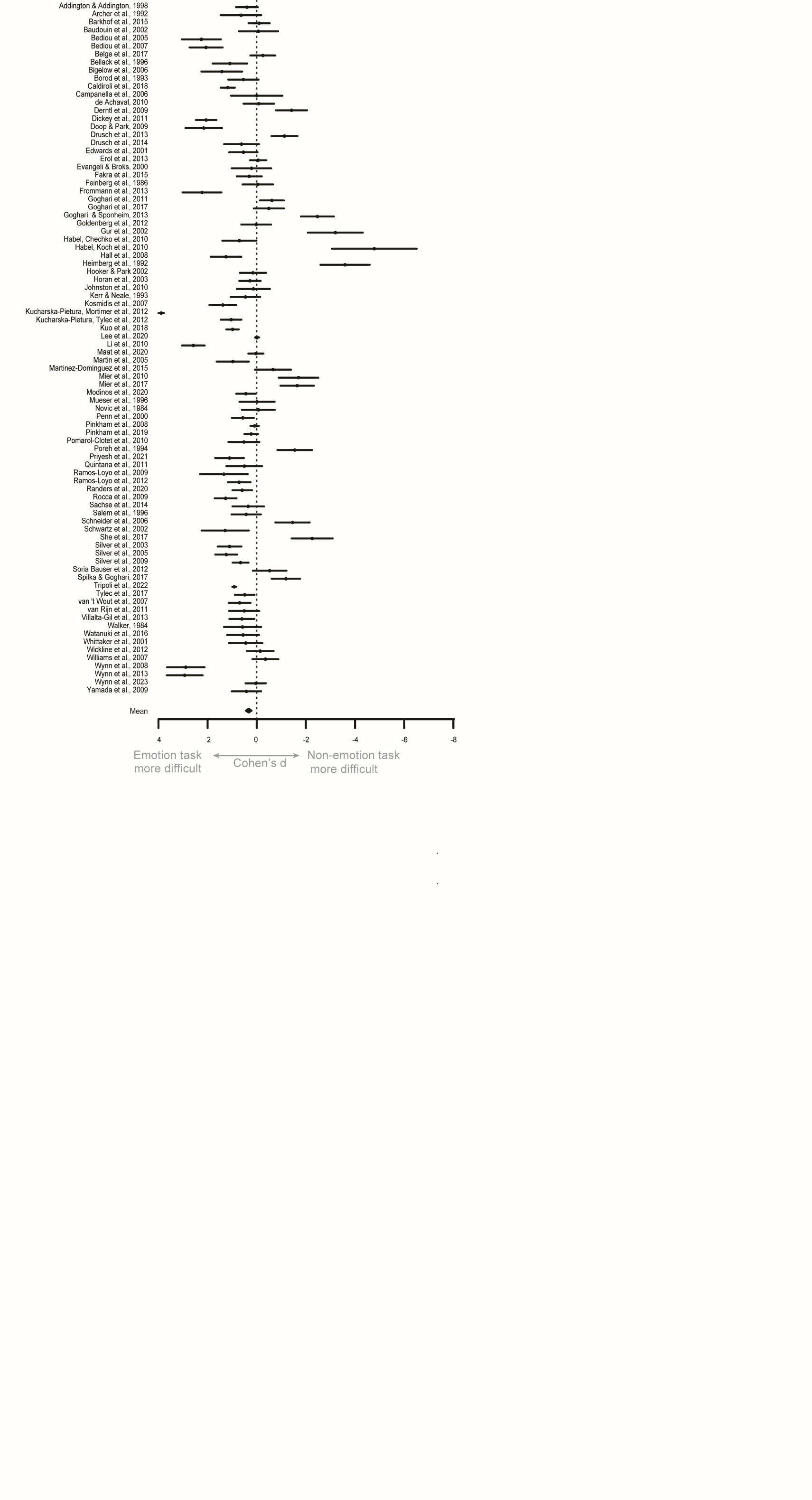
_**

*Note.* Effect sizes are presented comparing emotion and non-emotion task difficulty for each article. Error bars show 95% confidence intervals. Only a subset of articles had sufficient data to be included in this analysis.

**Fig. S11. Relationship between continuous task difficulty and the differential emotion impairment**

Continuous Task Difficulty

*Note.* Each effect size is represented by a bubble, weighted in size based on sample. The position of each bubble on the y-axis and x-axis indicates the magnitude of each effect size and value of the task difficulty moderator for that effect, respectively. The large confidence intervals in low-severity SSD samples reflect the small sample size and varied magnitude of deficits found across studies, likely due to sample diversity. In Fig 4C. the *Q* statistic tests the difference between emotion and non-emotion tasks. In Figs 4A., 4B., and 4D. the *Q* statistic tests the interaction between moderator variable and the type of task.

40 60 80 100

0 1 3

Cohen’s *d*

*Q*  = .69, *p* = .411

**S12. Publication bias analysis**

Our meta-regression dataset had substantial heterogeneity and dependency amongst effect sizes (i.e., multiple effect sizes coming from the same paper). As such, we used residuals (rather than raw effect sizes) of the combined model to produce a funnel plot (see Figure S12) and an extended multilevel meta-regression model to test for publication and time-lag biases (as per recommendations in Nakagawa et al.^2^). There was no evidence of small sample bias effects (slope = -.03, p = .938 95% CI = [-0.787, 0.729]). There was evidence of a significant time-lag bias (slope = -.02, p < .01, 95% CI = [-.03, -0.01]) indicating effect sizes reduced over time (see Figure S11). It is unlikely that true SSD-related deficits have reduced over time, so the time-lag bias indicates recent, more modest estimates may better reflect true deficits while estimates produced in older articles (and therefore our overall effect size) may be somewhat overestimated.

Alternative explanations for the time-lag bias effect were explored. First, we assessed whether the effect was explained by increasing sample sizes over time which can produce more precise and often smaller effect size estimates^3^. Consistent with this, there was a positive correlation between sample size and year of publication (*r* = .304, *p* <.001). However, the negative correlation between year of publication and effect size (*r* = -.342, *p* <.001) could not be explained by sample size as it remained stable when the effect of sample size was partialed out (*r* = -.337, *p* <.001). Next, we used Akaike Information Criterion to test whether the time-lag bias effect was caused by model over-fitting with the inclusion of additional variables. This was not the case as the model fit improved when including publication year (combined model AIC = 307.44 versus when testing for publication bias AIC = 294.50). As such, it appears the time-lag bias is a true effect not explained by increasing sample size over time or data overfitting. The time effect may be driven by more recent studies sampling low-severity SSD with more modest deficits.

When data was adjusted for time-lag bias, the magnitude of the overall SSD-related deficit was only slightly smaller (unadjusted Cohen’s *d* = .65, 95% CI [.56, .74]; adjusted Cohen’s *d* = .64, 95% CI [.54, .73]). As such, effect size estimates reported in the main text may be slightly over-estimated due to the impact of time-lag bias.

**
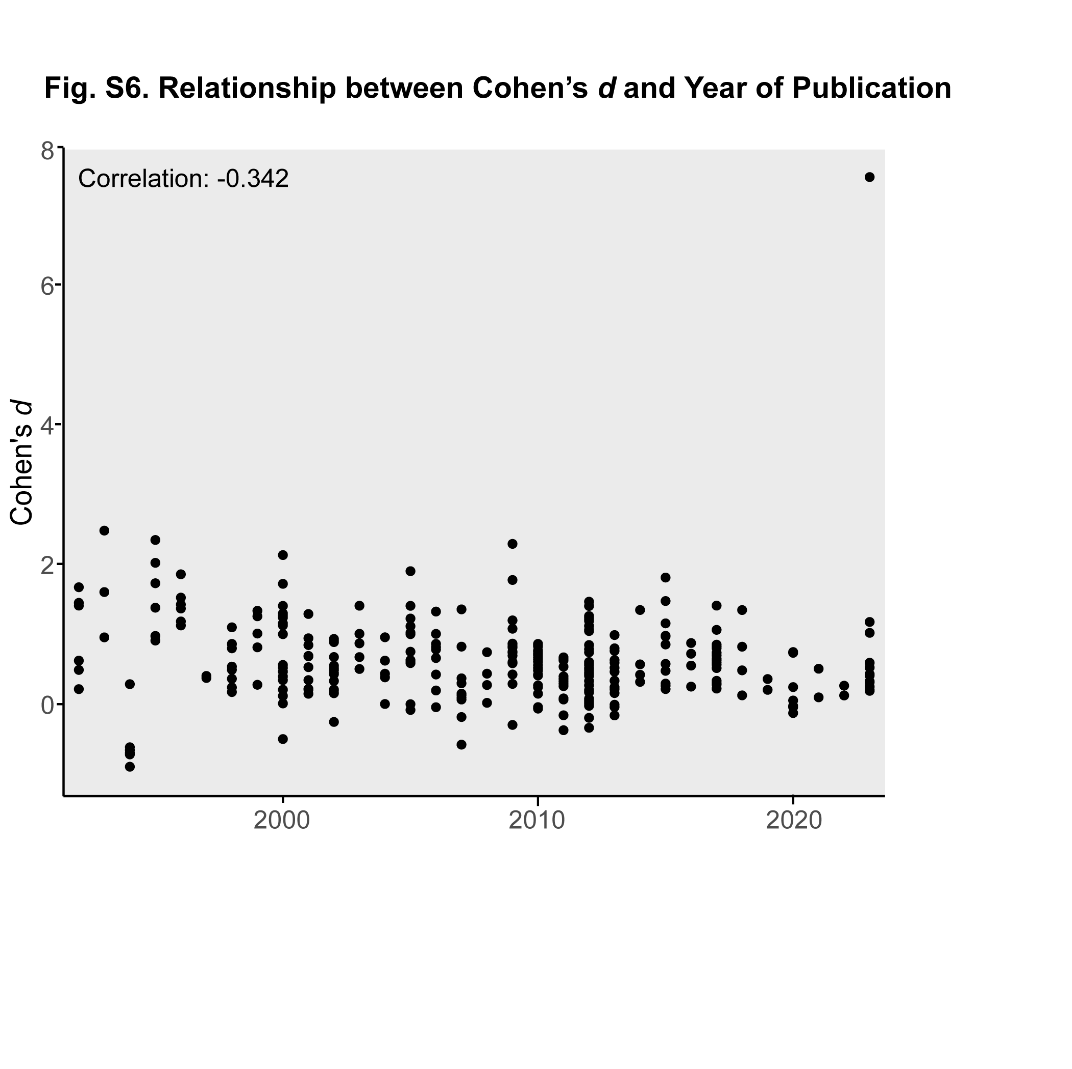
**

**Fig. S13. Relationship between Cohen’s *d* and year of publication**


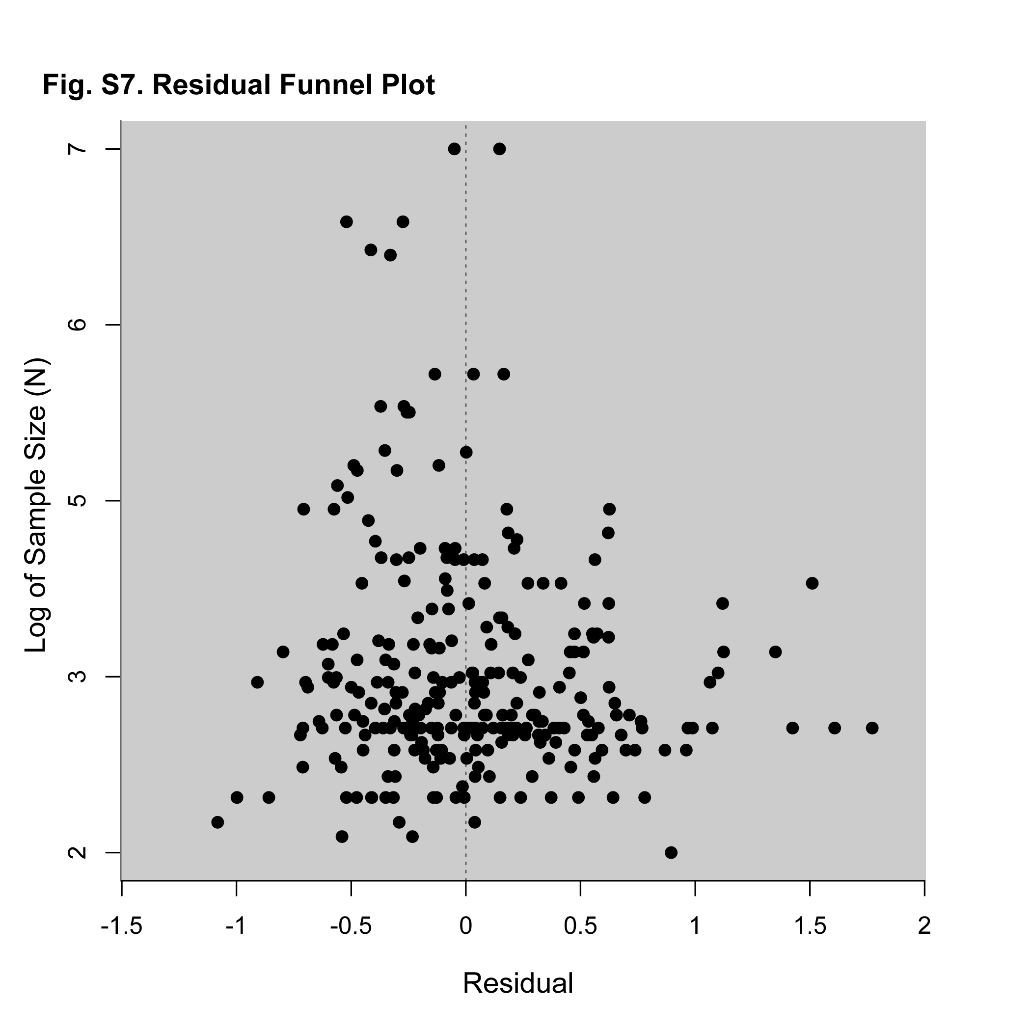


**Fig. S14. Residual Funnel Plot**

**References**

1. Foo YZ, Nakagawa S, Rhodes G, Simmons LW. The effects of sex hormones on immune function: A meta-analysis. *Biological Reviews*. 2017;92(1):551-571. doi:10.1111/brv.12243

2. Nakagawa S, Lagisz M, Jennions MD, et al. Methods for testing publication bias in ecological and evolutionary meta‐analyses. *Methods Ecol Evol*. 2022;13(1):4-21. doi:10.1111/2041-210X.13724

3. Jennions MD, Møller AP. Relationships fade with time: A meta-analysis of temporal trends in publication in ecology and evolution. *Proc Biol Sci*. 2002;269(1486):43-48. doi:10.1098/rspb.2001.1832
